# Long-Term Stability of Hydrothermally Aged and/or Dynamically Loaded One-Piece Diameter Reduced Zirconia Oral Implants

**DOI:** 10.3390/jfb14030123

**Published:** 2023-02-24

**Authors:** Ralf-Joachim Kohal, Anja Trinkner, Felix Burkhardt, Sebastian Berthold Maximilian Patzelt, Kirstin Vach, Monika Kušter, Anže Abram, Andraž Kocjan, Julian Nold

**Affiliations:** 1 Department of Prosthetic Dentistry, Center for Dental Medicine, Medical Center—University of Freiburg, Faculty of Medicine, University of Freiburg, Hugstetter Str. 55, 79106 Freiburg, Germany; 2 Institute of Medical Biometry and Statistics, Medical Center—University of Freiburg, Faculty of Medicine, University of Freiburg, Stefan-Meier-Str. 26, 79104 Freiburg, Germany; 3 Department for Nanostructured Materials, Jožef Stefan Institute, Jamova 39, 1000 Ljubljana, Slovenia

**Keywords:** one-piece, loading/aging, oral implants, zirconia implant, thermomechanical aging, phase transformation, fracture strength

## Abstract

The aim of this in vitro study was to evaluate the long-term stability of one-piece diameter reduced zirconia oral implants under the influence of loading and artificial aging in a chewing simulator as well as the fracture load in a static loading test. Thirty-two one-piece zirconia implants with a diameter of 3.6 mm were embedded according to the ISO 14801:2016 standard. The implants were divided into four groups of eight implants. The implants of group DLHT were dynamically loaded (DL) in a chewing simulator for 10^7^ cycles with a load of 98 N and simultaneously hydrothermally aged (HT) using a hot water bath at 85 °C. Group DL was only subjected to dynamic loading and group HT was exclusively subjected to hydrothermal aging. Group 0 acted as a control group: no dynamical loading, no hydrothermal ageing. After exposure to the chewing simulator, the implants were statically loaded to fracture in a universal testing machine. To evaluate group differences in the fracture load and bending moments, a one-way ANOVA with Bonferroni correction for multiple testing was performed. The level of significance was set to *p* < 0.05. In the static loading test, group DLHT showed a mean fracture load of 511 N, group DL of 569 N, group HT of 588 N and control group 0 of 516 N. The average bending moments had the following values: DLHT: 283.5 Ncm; DL: 313.7 Ncm; HT: 324.4 Ncm; 0: 284.5 Ncm. No significant differences could be found between the groups. Hydrothermal aging and/or dynamic loading had no significant effect on the stability of the one-piece diameter reduced zirconia implants (*p* > 0.05). Within the limits of this investigation, it can be concluded that dynamic loading, hydrothermal aging and the combination of loading and aging did not negatively influence the fracture load of the implant system. The artificial chewing results and the fracture load values indicate that the investigated implant system seems to be able to resist physiological chewing forces also over a long service period.

## 1. Introduction

As of today, due to its high load-bearing capacity and good osseointegration, as well as its biocompatibility, titanium can be considered the material of choice for oral implants [1,2,3,4,5]. Nevertheless, material related issues such as corrosion and wear, as well as growing patient demands in the area of biocompatibility and esthetics, have led to increasing research into non-metallic materials [6,7]. Those materials have been able to reach the market as potential alternatives to titanium [8]. Zirconium dioxide (=zirconia) ceramics as high-performance ceramics are increasingly becoming the focus of scientific attention [9]. The tooth-like color, immunobiological neutrality, corrosion stability and good mechanical properties are favorable properties of these materials [10]. Zirconia, which has been established in dental practice as a material for crown and bridge reconstructions, can also meet the requirements as an oral implant material. While Zirconia implants are already in clinical use [11,12,13], their market share is still small compared to titanium implants [14,15]. In addition, momentarily, there is only limited long-term evidence in the clinical application of zirconia implants in comparison to titanium implants [16,17]. Nevertheless, it is shown that the osseointegration, as well as early survival and success rates of zirconia oral implants are comparable to modern titanium oral implants [18,19]. Regarding soft tissue integration, obviously, materials from zirconia obviously show extremely positive biological behavior with connective tissue and epithelial cells with accelerated proliferation and healing processes [20,21]. While initial research focused mainly on osseointegration, current investigations concentrate on the mechanical properties of zirconia and the influence of hydrothermal and mechanical aging on the fracture load and long-term stability of those zirconia-based implants.

Zirconia, in its most common form, i.e., 3 mol%-yttria stabilized tetragonal zirconia polycrystal (3Y-TZP), is partially stabilized by yttrium oxide resulting in it being metastable in its tetragonal configuration at room temperature. The phase metastability is responsible for 3Y-TZP’s high strength and fracture toughness facilitating the stress-induced tetragonal-to-monoclinic (t–m) transformation toughening mechanism, where under applied stress the metastable tetragonal phase (grains) transforms to monoclinic. The aforementioned phase transformation results in a volumetric increase, which itself can limit and/or stop crack-propagation (t–m transformation toughening) [22]. Still, zirconia, to some extent (depending on the yttria content), has shown to be prone to the moisture-induced spontaneous t–m transformation in humid environments (known as low temperature degradation (LTD) or ageing). The LTD is said to contribute to the degradation of the material, starting at the surface, with the corresponding reduction in fracture load after hydrothermal aging [23,24]. It occurs when water molecules penetrate into the lattice structure of the material surface, causing a t–m transformation at the surface favoring the formation of microcracks which can lead to premature material fatigue [25]. Both phase transformation toughening and low-temperature degradation are based on the described t–m transformation. While the resulting compressive stress on the surface caused by a zone of transformation can favor loading characteristic [26,27], a steady increase in quantity of unfavorable microcracks caused by the LTD can disturb this balance and worsen loading characteristics [28].

However, recent investigations on the biomechanical stability of (regular diameter) zirconia implants could not show a consistent decrease in implant stability after hydrothermal loading [29]. The results of the different investigations were presenting data that led to the conclusion that these zirconia implants would sustain clinically over a long time period. However, no data are available on so-called reduced (narrow) diameter zirconia implants. In case of limited interdental or interimplant space, reduced diameter implants (RDI) may offer a solution to space deficits [30,31]. Furthermore, in cases with implant sites with narrow ridges, RDIs can be used not necessitating bone augmentation procedures [32,33]. While RDI titanium implants were evaluated in anterior [31,34] and posterior regions [35], there is a lack of studies evaluating zirconia RDIs. In contrast, regular diameter one-piece zirconia implants have been clinically evaluated with positive clinical and preclinical outcome [18,36,37].

Therefore, the aim of the present in vitro study was to evaluate a reduced diameter zirconia implants system regarding its long-term stability in an artificial chewing simulation using dynamic loading and/or hydrothermal aging. Furthermore, the fracture load was assessed in a universal testing machine.

## 2. Materials and Methods

### 2.1. Study Implants and Experimental Setup

**Figure 1:**
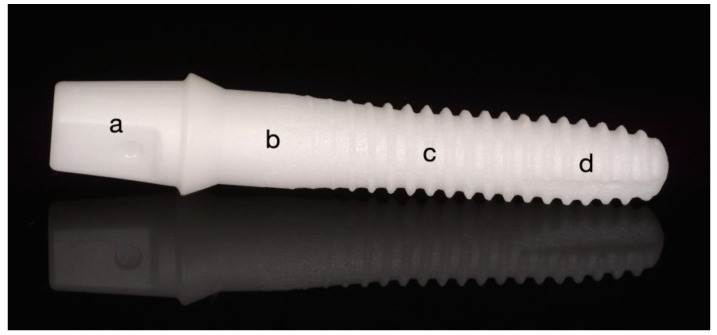
The one-piece zirconia implant ZiBone^®^: (**a**) conical abutment part, (**b**) transmucosal area, (**c**) endosseous cylindric-conical part, (**d**) bone chip reservation groove.

A total of 32 reduced-diameter one-piece implants fabricated from 3Y-TZP with an endosseous diameter of 3.6 mm and an endosseous length of 14.5 mm (ZiBone^®^, COHO Biomedical Technology CO, Taoyuan City, Taiwan; sterilized by autoclaving, Figure 1) were used for the experiment. The zirconia implants were fabricated via injection molding. Subsequently, they were sintered and ground to the final shape and the endosseous part was sandblasted using zirconia sand resulting in an Ra of approximately 5 µm. The zirconia implants used for the four groups were all prepared in the same condition and, thus, standardized (manufacturer’s information: sintering conditions: 1350 °C for 2 h; powder composition: ZrO_2_ + HfO_2_ + Y_2_O_3_: ≥99%, Y_2_O_3_: >4.5% ≤6.0%, HfO_2_: ≤5.0%, Al_2_O_3_: ≤0.5%, other oxides: ≤0.5%; bulk density: 6.04 g/cm^3^, grain size: 0.365 μm and 4-point flexural strength: 919.14 MPa). The implants had a conical abutment with a height of 5 mm to seat a crown and a cylindric-conical body design with a bone chip reservation grove at the bottom. The transmucosal height amounted to 4 mm. For the present investigation, the implants were divided into four groups at eight implants each: group DLHT; group DL; group HT and control group 0 (Figure 1).

**Figure 2:**
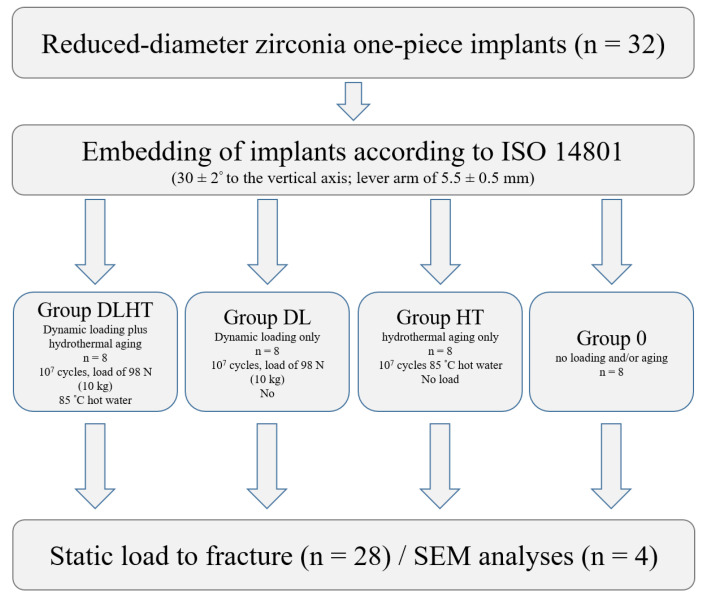
Set-up of the experiment. Seven specimens per group were intended to be exposed to the static load until fracture; one specimen per group was intended for surface microstructural and subsurface phase and microstructural characterization.

The implants of group DLHT were both dynamically loaded and hydrothermally aged, the implants of group DL were only dynamical loaded and the implants of group HT were only hydrothermally aged in a chewing simulation device (CS-4.8; SD Mechatronik, Feldkirchen-Westerham, Germany), while the implants of control group 0 were neither hydrothermally aged nor dynamically loaded. One implant of each test group was used for the detailed phase and microstructural evaluation. All other implants were loaded to fracture in a static loading test (Zwick Z010/TN2S, Zwick Roell, Ulm, Germany) and the results were statistically analyzed (Figure 2).

### 2.2. Preparation of the Test Specimens

**Figure 3:**
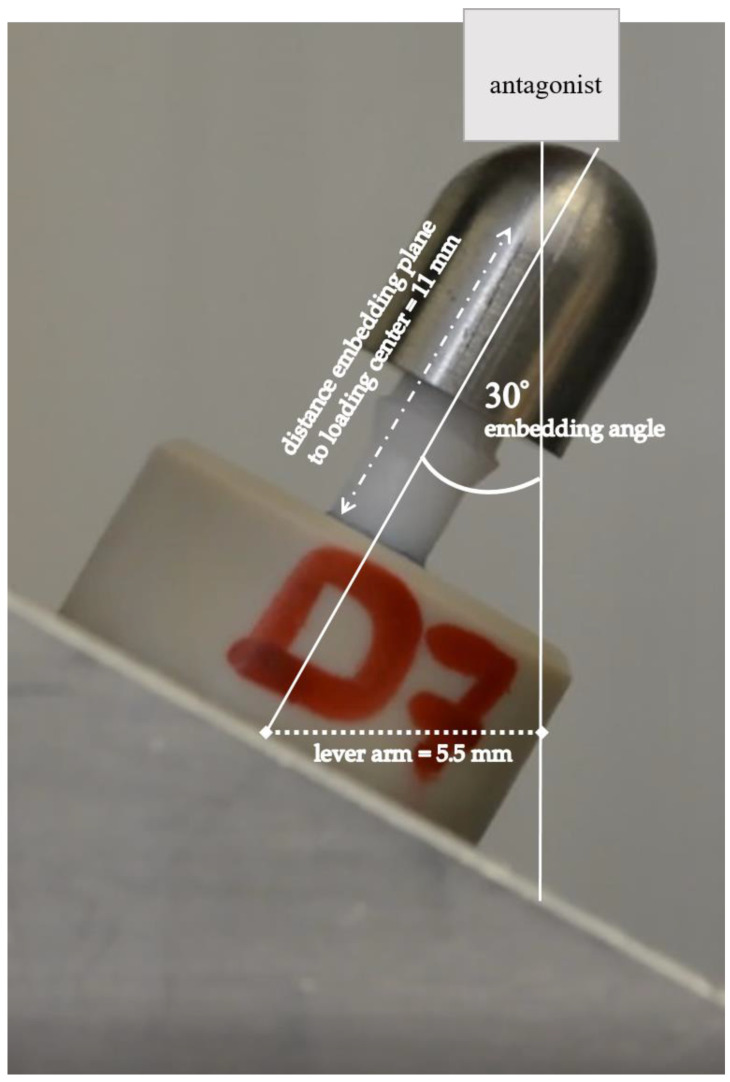
Investigated one-piece zirconia implant embedded according to the ISO 14801 standard with a dual-curing composite in a PEEK tube and a loading hemisphere attached.

All implants were embedded in accordance with the ISO 14801 standard following the protocol described by Spies at al. [38]. The implants were inserted into PEEK tubes and stabilized with a dual-cure composite (LuxaCore Automix Dual, DMG, Hamburg, Germany). The angle of implant embedding was 30 (±2)° to the vertical axis. The distance between the point where the implant exited the composite material and the loading center on the stainless-steel loading hemisphere was 11 (±0.5) mm. A lever arm of 5.5 (±0.5) mm resulted from this embedding. For the simulation of the peri-implant bone recession of 3 mm, the implant’s endosseous part was exposed above the composite material level (Figure 3).

### 2.3. Hydrothermal Aging and/or Dynamic Loading

The implants of group DLHL and DL were installed into a computer-controlled chewing simulator (CS-4.8) and were exposed to 10,000,000 chewing cycles with a load of 98 N (10 kg) at a 30° loading angle. The opposing stainless-steel antagonist had a plane loading surface that contacted the loading hemisphere attached to the implants. A loading cycle consisted of a vertical (2 mm) and subsequent horizontal (0.5 mm) movement component to imitate the natural chewing in humans. The speed for the vertical movement was 60 mm/s and the one for the horizontal movement was 55 mm/s. The frequency of a cycle was 1.27 Hz. To achieve hydrothermal aging (groups DLHT and HT), the test chamber of each implant was filled with distilled water and kept at a temperature of 85 °C. The implants of group HT were placed in the hot water chambers and left there for the 10 million chewing cycles without the loading component. The samples of the different groups DLHT, HT and DL were inspected twice a day for the whole duration of the project for adverse events.

After dynamic loading, one implant each of group DLTH, DL, HT and 0 was investigated for the effects that the different loading/aging procedures had on the implant microstructure structure.

### 2.4. Surface Microstructural Characterization

The representative surface morphologies of the transmucosal area and endosseous cylindric-conical part of the implants were imaged at various magnifications using field-emission scanning electron microscopy (FE-SEM) (Supra 35 LV, Carl Zeiss AG, Oberkochen, Germany). High-magnification micrographs were taken to analyze the microstructural features of the implant surface before and after the thermomechanical treatment. For the imaging of the non-coated sample surface accelerating voltages between 2 and 5 kV were employed.

### 2.5. Subsurface Phase and Microstructural Composition

To obtain the phase composition and changes near surface of the 3Y-TZP specimens, the grazing-incidence X-ray diffraction technique, a small-angle X-ray scattering technique, was used to obtain lattice planes near perpendicular to the specimen surface [39]. The Malvern Panalytical Empyrean X-ray diffractometer with Cu-target tube (λKα1 = 0.15406 nm and λKα2 = 0.154439 nm) and configuration Chi-Phi-x-y-z stage, theta/theta geometry was used. The measurements were collected using a parallel plate collimator 0.27 on the diffracted beam path and hybrid monochromator 2xGe (220) for Cu on the incident beam path in a range of 27°–38° 2Θ, with a step size of 0.02°. The GI-XRD data were analyzed by HighScore Plus XRD Analysis Software database PDF-4+. The relative amount of the transformed monoclinic zirconia (m-ZrO_2_) on all surfaces was determined from the integral intensities of the monoclinic ($\bar{1}$ 1 1)m and (1 1 1)m, and the tetragonal (1 0 1)t peaks according to the method of Garvie and Nicholson. This method is the most commonly applied to determine the phase composition of zirconia powders and compacts with randomly distributed m-ZrO_2_ and t-ZrO_2_ phases at any distance from the surface exposed to the XRD analysis.

Subsurface microstructural changes after the thermomechanical treatment were analyzed with a Helios NanoLab 650 Focused Ion Beam Scanning Electron Microscopy (FIB-SEM) (FEI, Hillsboro, OR, USA) on the ion-milled cross-sections. A 0.5-mm-thick platinum coating was deposited onto the surface of interest using an ion-beam-assisted gas-injection system at 30 kV and 0.43 nA to minimize the extensive curtaining effect. Across the selected interface regions, FIB milling was carried out using an ion beam at 30 kV and 65 nA, followed by ion polishing at 30 kV and 21 nA. Subsurface areas were exposed and observed in situ at an angle of 52°, using an electron probe at 3–10 kV and 40–80 pA.

### 2.6. Static Loading Test in a Universal Testing Machine

With the exception of the implants that have been used for microstructural analysis (n = 1 per group), all other implants that survived the dynamic loading procedure in the artificial chewing simulator, were loaded until fracture in a universal testing machine (Z010/TN2S, ZwickRoell, Ulm, Germany). The compressive load was executed at an angle of 30° and a cross-head speed of 10 mm/min. The maximum load (Fmax) to fracture was recorded using the software (testXpert^®^ V7.1, ZwickRoell) and an XY-writer (Spare 2000, Kipp&Zonen, Delft, The Netherlands).

### 2.7. Statistical Analysis

For the descriptive analysis, medians, means, standard deviations, minimum and maximum were calculated and presented in boxplots. A one-way ANOVA was applied to compare group differences in the fracture load and bending moment. Subsequently, pairwise comparisons were performed and the Bonferroni method for correcting for multiple testing was used. The statistical significance level was set to *p* < 0.05. Calculations were performed using the statistical software STATA 17.0 (StataCorp, LP, College Station, TX, USA).

## 3. Results

### 3.1. Results of the Dynamic Loading Test

**Figure 4:**
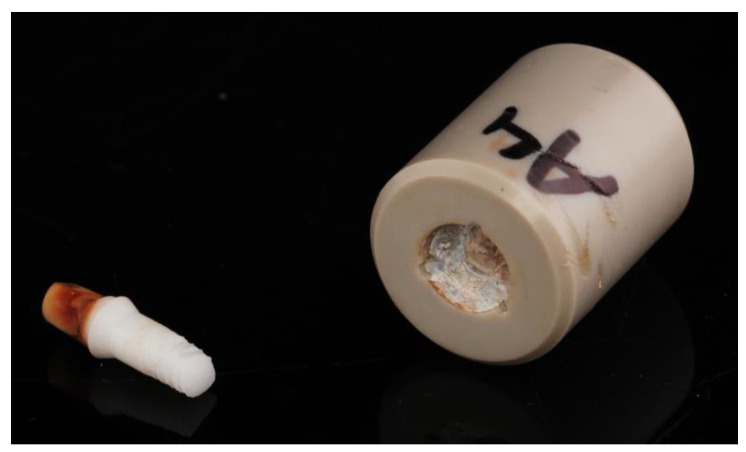
Fractured implant (DLHT4) after dynamic loading and hydrothermal aging. The fracture occurred inside the tube.

After completion of the dynamic loading and/or hydrothermal aging in the chewing simulator, the test specimens were evaluated for any adverse events. In one specimen, it was observed that the implant had fractured inside the embedding material (DLHT4). Since this adverse event was not seen during the inspections of the tests, it was not possible to determine the timepoint of the fracture. Figure 4 shows the failed test specimen. All other specimens survived the dynamic loading and/or aging without failure. This resulted in a survival rate of 87.5% for group DLHT and a survival rate of 100% for the two other groups (DL, HT). In total, a survival rate of 96% was observed.

### 3.2. Scanning Electron Microscopy Analysis

**Figure 5:**
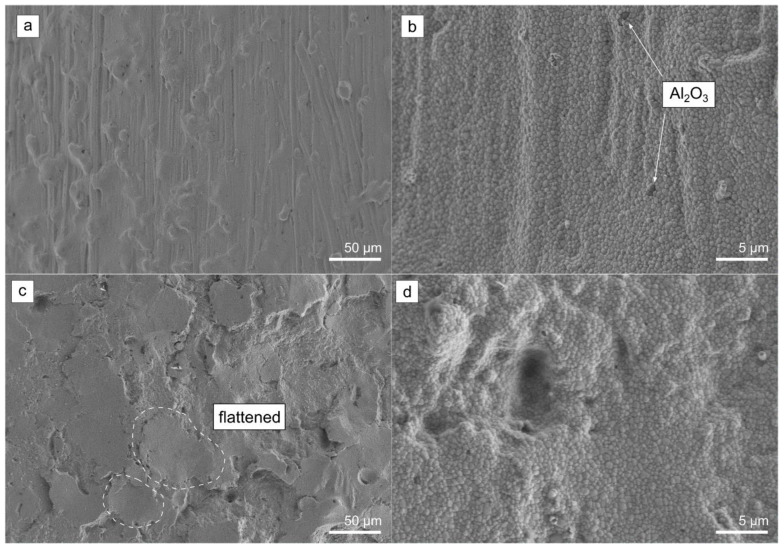
FE-SEM micrographs of the control group 0 implants (**a**,**b**) transmucosal and (**c**,**d**) endosseous cylindric areas. Arrows indicate isolated alumina grains in the zirconia matrix. The circled areas are showing flattened areas as a consequence of sandblasting.

The surface topographies of the implant‘s transmucosal (Figure 1b) and endosseous cylindric (Figure 1c) areas were investigated by FE-SEM (Figure 5). On the transmucosal area, surface irregularities in the form of vertical shallow grooves intersected with bulges and dimples were observed, which were presumably originating from the implants injection (de)moulding process (Figure 5a). Higher magnification micrograph revealed a dense microstructure consisting of submicron-sized 3Y-TZP grains (Figure 5b). In their matrix, few alumina grains were also observed, typical for the 3Y-TZP materials containing small amount of alumina [40]. The endosseous cylindric area on the other hand showed a more rougher surface area with deeper dimples and flattened areas as a consequence of “softer” sandblasting with zirconia sand resulting in an apparent Ra of approximately 5 µm (Figure 5c). The higher roughness as a result of sandblasting can be clearly observed in higher magnification micrograph showing high level of surface irregularities (Figure 5d).

**Figure 6:**
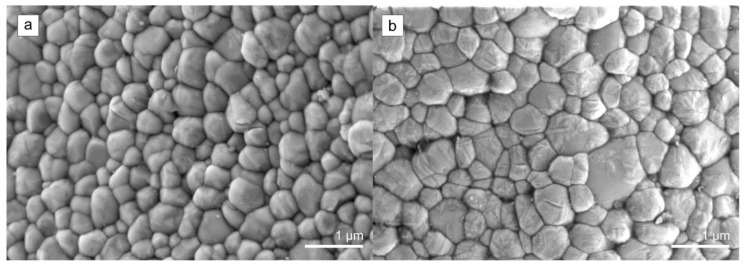
High-magnification FE-SEM micrographs showing microstructure of the (**a**) control group 0 implant specimen and (**b**) after thermomechanical ageing (DLHT).

High magnification FE-SEM micrographs were taken to analyze the microstructure of the control group implant specimen and after thermomechanical ageing (DLHT) (Figure 6). The microstructure of the control group 0 specimen was dense and uni-modal, exhibiting equiaxed, polygonal grains with no pore inclusions (Figure 6a). The microstructure of the DL sample was similar. However, distinct changes were observed in specimens that underwent hydrothermal ageing (HT and DLHT). Figure 6b is showing the microstructure of the DLHT specimen. In contrast to smooth grains in the control group, self-accommodating martensitic variants of various orientations are visible that either intersect within a single grain, or they grow continuously from grain to grain, as was commonly observed elsewhere [41]. In addition to monoclinic twinning, microcracks visible on the grains were observed, but no trans/intragranular grain fractures nor grain-pull outs [42].

### 3.3. Subsurface Phase and Microstructural Composition

**Figure 7:**
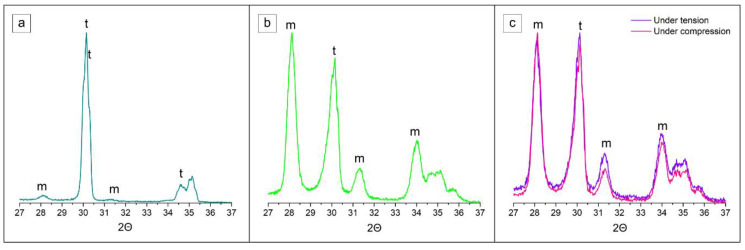
Representative grazing-incidence X-ray patterns of the (**a**) as-received control group 0 implant and after (**b**) hydrothermal ageing (HT) and (**c**) dynamic loading plus hydrothermal ageing (DLHT). t-ZrO_2_ and t-ZrO_2_ phases are labelled with letters m and t, respectively.

Grazing-incidence XRD analysis of the implant specimens was employed in order to extract information about the phase composition and changes on the uneven and rough implant surface from the transmucosal area (Figure 1). The tetragonal zirconia (t-ZrO_2_) phase was the major constituent of the control group specimen (Figure 7a). In addition, traces (3.9%) of the monoclinic (m-ZrO_2_) phase were detected already prior thermomechanical loading. No significant changes were observed in diffractograms from DL specimen. Hydrothermal ageing triggered LTD, in accordance with FE-SEM observations (Figure 7b), where the estimated amount of transformed monoclinic phase was significant, i.e., 55.7%. Similarly, the dynamically loaded and hydrothermal aged (DLHT) group was exposed to t–m transformation with an estimated 53.4 % of monoclinic phase, which was recorded at the tensile side of the loading force (Figure 3). In addition, the measurement was also performed at the compressive side of the loading. A slightly lower value of 52.5% was recorded.

**Figure 8:**
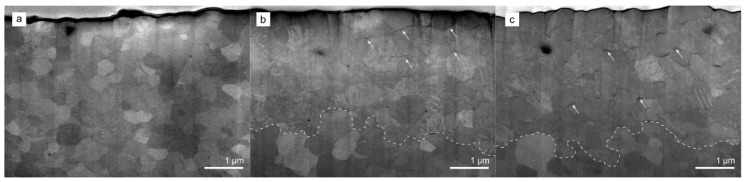
SEM-FIB micrographs showing ion-milled cross-sections of implant specimens: (**a**) control group 0, (**b**) hydrothermally aged (HT) and (**c**) dynamically loaded and hydrothermally aged (DLHT). White arrows and dotted lines indicate the formation of martensitic variants and microcracks, respectively. The transition to the unaffected bulk is indicated by the white dashed line.

The SEM-FIB analysis provided an insight into microstructural changes taking place at the immediate sub-surface region of the implants after the thermomechanical ageing experiment (Figure 8).

The microstructure of the control group specimens was typical of 3Y-TZP consisting of equiaxed grains differing in color due to the orientation contrast (Figure 8a). The first layer of surface grains indeed suggested hints of martensitic variants, as indicated, that were also observed with the FE-SEM (Figure 6a). Hydrothermal ageing promoted the LTD process accompanied with the t–m transformation, which can be clearly discerned from Figure 8b. The grains are intersected with twin-related monoclinic variants with lath-like features, which are positioned predominantly perpendicular to the surface. In addition, few intergranular microcracks oriented parallel to the surface can be observed in the transformed subsurface layer, which thickness appears to be up to ~3–4 μm (Figure 8b). The transformed layer is homogeneous, resulting in a fairly clear and coherent boundary between transformed and untransformed subsurface layer. Similar subsurface changes were observed in the DLHT specimens. In accordance with XRD analysis (Figure 8b,c), the transformed layer was of comparable thickness of ~3–4 μm. Likewise, the transformed layer contained twin-related monoclinic variants. However, it appeared that the microcracking in the layer was more extensive owing to dynamic loading.

### 3.4. Static Loading Test until Fracture

**Figure 9:**
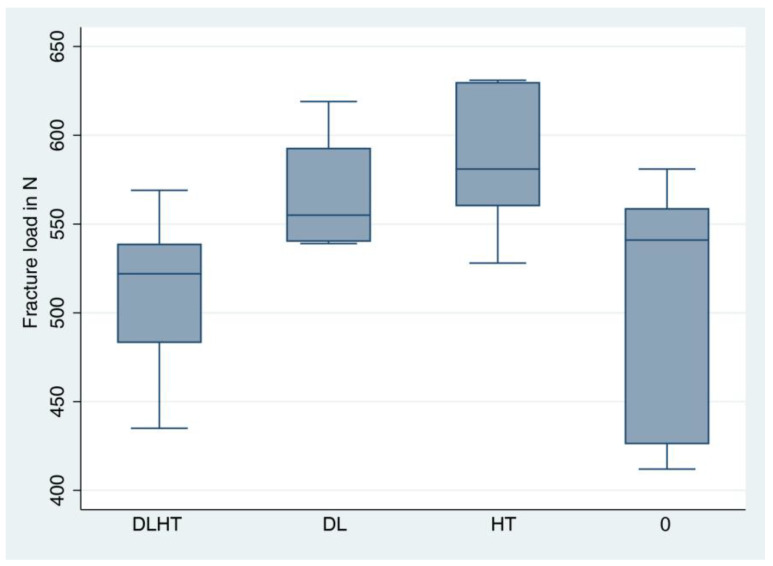
The calculated fracture load is visualized with boxplots (see Table 1 for detailed data). A whisker shows all samples which are within 1.5 times of the interquartile range, all other data are plotted as outliers.

**Figure 10:**
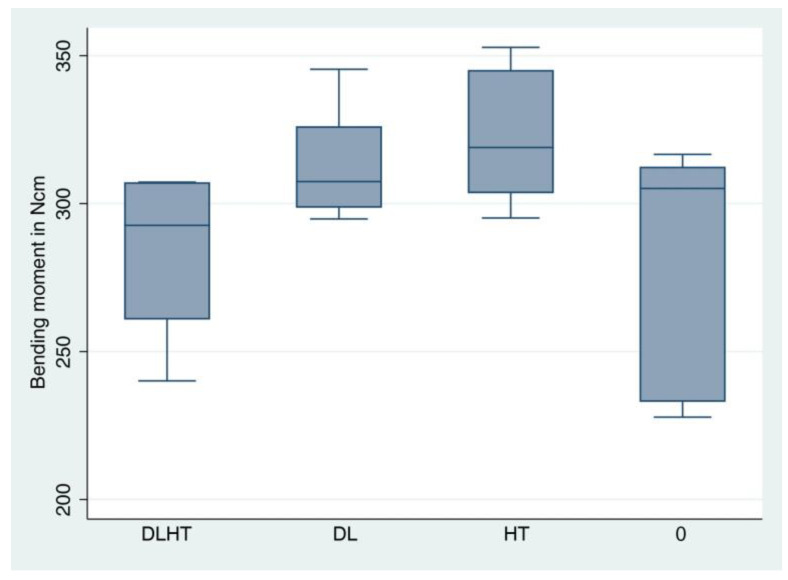
The calculated bending moment is visualized with boxplots (see Table 1 for detailed data). A whisker shows all samples which are within 1.5 times of the interquartile range, all other data are plotted as outliers.

Seven test specimens from each of the groups DL, HT and control group 0 and six from group DLHT (DLHT4 had to be excluded since it fracture during the artificial loading test) were exposed to the static fracture load test in the universal testing machine. The fracture load values amounted to 512 N for group DLHT, to 569 N for group DL, to 588 N for group HT, and to 516 N for control group 0 (Figure 9, Table 1,). The values for the average bending moments were 283.5 Ncm for DLHT, 313.7 Ncm for DL, 324.4 Ncm for HT and 284.5 Ncm for 0 (Figure 10, Table 1).

ANOVA revealed a statistically significant difference between all groups (*p* = 0.016). However, after pairwise comparison, no significant differences could be found between the groups anymore.

### 3.5. Fracture Analysis

**Figure 11:**
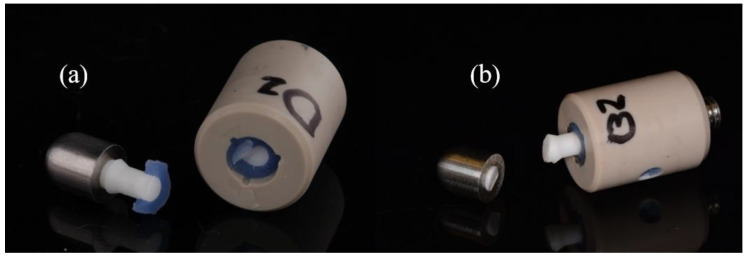
The different implant fracture localizations after static loading: (**a**) fracture occurred inside the composite material in the tube, (**b**) the only fracture at the abutment level.

In total, 26 of the implant fractures occurred inside the composite material. Only one specimen (DL2) fractured in the area of the abutment. Figure 11 shows the two different fracture localizations.

## 4. Discussion

The present in vitro study evaluated the long-term stability of a reduced-diameter one-piece zirconia implants when dynamically loaded and hydrothermal aged in a chewing simulator. So far, there is a lack of data on the mechanical long-term behavior of reduced-diameter zirconia implants in the literature [43].

The present investigation could find that hydrothermal aging and/or dynamic loading had no statistically significant effect on the stability of the one-piece diameter-reduced zirconia implants. The absolute fracture loads/bending moments indicate that the investigated implant system seems to be able to resist physiological chewing forces long-term.

The static loading until fracture revealed a mean bending moment of 283.5 Ncm for group DLHT, 313.7 Ncm for group DL and 324.4 Ncm for group HT. The values for DL and HT were slightly higher than those of the unloaded specimens (control group 0) at 284.6 Ncm. However, no statistically significant differences have been found in pairwise comparison, it could be stated within the limits of the investigational set-up, that there is a tendency that the dynamic loading only and the hydrothermal aging only had a positive influence on fracture load and bending moment. The simultaneous dynamic loading and hydrothermal aging, however, in combination, exerted neither a negative nor positive effect on the bending moment of the investigated implants. The positive influence of the hydrothermal ageing on the strength of 3Y-TZP was already reported [27,44,45] and was ascribed to the compressive stresses that evolve in the transformed layer (Figure 8b) due to the volume increase in t–m transformation compensating for the tensile stresses during the strength testing. Furthermore, Sanon et al. (2013) have shown, among others, that after aging [low-temperature degradation (LTD) with also a t–m transformation] for durations compatible with clinical use, 3Y-TZP with porous surface presented a higher fatigue performance [23,46].

However, the combined influence of loading and hydrothermal aging did not result in an increase in stability compared to loading only and hydrothermal aging only, which was also shown in the study of Tinschert et al. [47]. The combination of loading and hydrothermal aging resulted in a “drop” of the mean bending moment for the DLHT group, but was not inferior to the control group 0. This was indicative on the annihilation of the beneficial compressive residual stresses in the t–m transformed layer of the HT specimen (Figure 8b). The transformed layer of the DLHT was indeed different, containing a higher amount of microcracks (Figure 8c). More work is needed, however, to study and properly quantify the observed differences.

Regarding implant diameter and fracture stability, Bethke and coworkers (2020) systematically evaluated the literature on fracture resistance of one- and two-piece zirconia oral implants in vitro [48]. They differentiated implant diameters from 3.0 to 3.3 mm, 3.8 to 4.4 mm and 4.5 to 5.0 mm. The authors did not find a statistically significant difference for the bending moment at fracture regarding the different implant diameters ranging from 3 to 5 mm for one-piece implants. Kammermeier et al. (2016) evaluated nine one-piece narrow-diameter (3.3 mm) implants after long-term thermal cycling and mechanical loading [49]. The authors reported that a mean fracture force of 281.7 N led to fracture of the implants (bending moment of 215 Ncm). In the same investigation, Kammermeier et al. found for a 3.8 mm diameter one-piece implant a mean load leading to fracture of 440.4 N (calculated bending moment of 336 Ncm) [49]. Therefore, it could be stated that the 3.6 mm diameter implants of the present investigation fit well between the results of 3.3- and 3.8-mm diameter implants.

A comparable investigation to the present investigation was executed by Burkhardt et al. (2021) [50]. The evaluated reduced diameter implants showed an average fracture load of 628 N and a bending moment of 349 Ncm. The authors concluded that fracture load values of those implants suggest a reliable intraoral clinical application in the anterior jaw regions. However, somewhat larger in diameter, the implants of the present investigation showed a lower bending moment than the 3.4 mm of the former investigation.

Nevertheless, the observed bending moment of the loaded and aged zirconia implant (283.5 Ncm) was higher than bending moments measured in vivo [51,52]. When we take, according to Bethke et al. (2020), the highest bending moment measured in vivo (95 Ncm) and apply a safety buffer of 100%, and consider a minimum fracture resistance of 200 Ncm sufficient to guarantee clinical safety, the present reduced-diameter implant system meets this demand [48,51].

The results of the fracture resistance from the review of Bethke et al. (2020) that no statistically significant difference regarding fracture stability for implant diameters ranging from 3 to 5 mm are obviously not back-upped clinically [48]. In the systematic review with meta-analysis of Roehling et al. (2018), the authors found that a total of 22 zirconia implants were reported to have fractured (1.95%) [16]. Of these 22 implants, 15 had a diameter of 3.25 mm, 4 had a diameter of 4.0 mm and no information was given on the diameter of the remaining 3 implants. The fracture incidence of zirconia implants was clearly associated with a decreasing implant diameter [53].

The limitations of the present investigation lie in the characteristics of an in vitro design. It can only approximate clinical reality since it is only a simulation of nature and not all clinical variables can be exactly applied (exact chewing simulation, temperature changes, biomechanical prerequisites and more). One limitation of the present investigation is also the low number of test samples. However, many other investigations have also been presented with a rather low sample size [48,51]. Nevertheless, this type of investigation may give a hint of whether an implant made from zirconia possibly may resist physiological forces. So far, the performed in vitro investigations [48,51] seem to support this assumption.

It would be a progress if the artificial chewing simulations could be even more closer to reality than they are at the moment. The development of such simulators might be a further research avenue for those interested in.

## 5. Conclusions

Within the limitations of the present investigation, it can be concluded that neither dynamic loading nor artificial hydrothermal aging, nor the combination of both, could statistically significantly influence the fracture load/bending moment of the investigated reduced diameter implant system. The revealed fracture load/bending moment of all groups was higher than the average masticatory forces expected in vivo. The investigated implant appears, therefore, to be suitable for long-term clinical use. Hydrothermal ageing triggered the t–m transformation, whereas this was not seen with dynamic loading alone. SEM-FIB micrographs showed the formation of martensitic variants (twinning). However, this transformation did not influence the stability of the implants negatively.

## Figures and Tables

**Table 1:** Fracture load (in N) and bending moment (in Ncm) of the different groups after testing in the universal testing machine.

Group		Fracture Load [N]	Bending Moment
DLHT (dynamic loading & hydrothermal aging)	Median	522	292.7
	Mean	512	283.5
	SD	47	279
	Minimum	435	240.1
	Maximum	569	307.3
DL (dynamic loading & no hydrothermal aging)	Median	555	307.4
	Mean	569	313.7
	SD	30	178
	Minimum	539	294.8
	Maximum	619	345.4
HT (no dynamic loading & hydrothermal aging)	Median	581	319.0
	Mean	588	324.4
	SD	39	222
	Minimum	528	295.1
	Maximum	631	352.8
0 (no dynamic loading & no hdydrothermal aging)	Median	541	305.1
	Mean	516	284.5
	SD	67	378
	Minimum	412	227.8
	Maximum	581	316.6

## Data Availability

The datasets generated and analyzed during the current study are available from the corresponding author on reasonable request.

## Abbreviations

ISO	International Organization for Standardization
DLHT	implants dynamically loaded and hydrothermally aged
DL	implants only dynamically loaded
HT	implants only hydrothermally aged
3Y-TZP	3 mol%-yttria stabilized tetragonal zirconia polycrystal
LTD	low temperature degradation
RDI	reduced diameter implants
FE-SEM	field-emission scanning electron microscopy
GI-XRD	grazing-incidence X-ray diffraction
FIB-SEM	focused Ion Beam Scanning Electron Microscopy
